# Research progress on the damage of lipid peroxidation to the body and its correlation with metabolic diseases

**DOI:** 10.3389/fmolb.2026.1793466

**Published:** 2026-03-02

**Authors:** Jiali Wang, Hongli Li, Yuhan She, Chongli Xu, Kun Peng, Wenqiao Ding

**Affiliations:** 1 Department of Medicine and Food, Laiwu Vocational and Technical College, Jinan, China; 2 College of Medical Technology, Chongqing Medical and Pharmaceutical College, Chongqing, China; 3 College of Biology and Food Engineering, Jilin University of Chemical Technology, Jilin, China

**Keywords:** antioxidation, free radical, lipid peroxidation, metabolic disease, oxidative stress

## Abstract

Lipid peroxidation is a critical oxidative stress response implicated in the pathogenesis of numerous metabolic diseases, including cardiovascular diseases, diabetes, and non-alcoholic fatty liver disease (NAFLD). While its role in damaging cellular components such as membranes, proteins, and DNA is well-documented, a significant translational gap persists between our mechanistic understanding and the development of effective clinical interventions. This review critically examines this disconnect by proposing and applying a three-tiered analytical framework. First, we identify and compare the hierarchy of initial molecular targets (e.g., mitochondrial cardiolipin, LDL phospholipids, specific protein thiols) across diseases, arguing that this hierarchy dictates pathological specificity. Second, we trace how these initial insults propagate through shared yet context-dependent mechanistic themes—metabolic dysregulation, inflammatory amplification, and cell death decisions—to drive organ-specific pathology. Third, we synthesize and critically evaluate current and emerging therapeutic strategies (e.g., antioxidants, ferroptosis inhibitors, nutritional modulation) against this mechanistic backdrop, highlighting their potential, limitations, and the need for mechanism-informed, personalized approaches. By moving beyond a descriptive catalog of effects, this review aims to provide a dynamic, intervention-oriented perspective essential for bridging basic science discoveries with translational innovation in combating lipid peroxidation-associated metabolic disorders.

## Introduction

The escalating global burden of metabolic diseases underscores an urgent need to refine our understanding of their underlying pathological drivers. Oxidative stress, and lipid peroxidation in particular, has been firmly established as a common thread linking diverse conditions such as diabetes, atherosclerosis, NAFLD, and neurodegenerative disorders (Queathem et al., 2025; García-Pastor et al., 2024). Decades of research have meticulously cataloged the reactive species involved, the resulting oxidation products (e.g., MDA, 4-HNE), and their detrimental effects on cellular structures and functions (Garbsch et al., 2024; Ma et al., 2025). However, this wealth of descriptive knowledge has not readily translated into breakthrough therapies targeting lipid peroxidation. Clinical trials of broad-spectrum antioxidants have yielded largely disappointing results, revealing a fundamental disconnect between simplistic ‘free radical scavenging’ concepts and the nuanced reality of disease pathogenesis (Saimoto et al., 2025).

This review is motivated by a central, unresolved question: How can our detailed mechanistic knowledge of lipid peroxidation be leveraged to design effective, context-specific therapeutic strategies?

To address this, we argue that the paradigm must shift from organ-centric description to a mechanism-prioritized framework. The core of our argument is that organ specificity in peroxidation-driven pathology arises not from the nature of the final peroxidation products, but from an inherent hierarchy of initial molecular targets dictated by tissue-specific redox landscapes and metabolic vulnerabilities. To systematically explore this thesis, we propose and apply a three-tiered analytical framework: (1) The Molecular Initiation Tier: A comparative hierarchy of initial targets (e.g., specific lipids or proteins) that determines disease entry points. (2) The Pathological Propagation Tier: A set of cross-cutting mechanistic themes (e.g., bioenergetic failure, sterile inflammation, regulated cell death) through which the initial insult amplifies into organ dysfunction. (3) The Intervention Tier: A critical appraisal of strategies that must be aligned with the specific mechanisms active in each tier and disease context.

Herein, we synthesize recent advances through this lens. We first revisit the core chemistry of lipid peroxidation and its products, then establish the concept of molecular target hierarchy. Crucially, unlike conventional reviews, we abandon a disease-by-disease organ-based narrative. Instead, the core sections are organized around the aforementioned cross-cutting propagation themes (Metabolic Dysregulation, Inflammatory Amplification, Ferroptosis), using specific diseases as exemplars to illustrate how the initial target hierarchy funnels into these shared pathways. We critically integrate our own research using a transgenic NPC1L1-overexpressing pig model of NAFLD as a case study to demonstrate how a defined metabolic insult propagates via lipid peroxidation. Finally, we evaluate therapeutic strategies not as a list of options, but as interventions that must be mapped onto specific nodes within this mechanistic cascade. By adopting this integrated perspective, we aim to move the field from cataloging damage towards constructing a predictive framework that can guide targeted intervention.

### Lipid peroxidation: core chemistry and product

**FIGURE 1:**
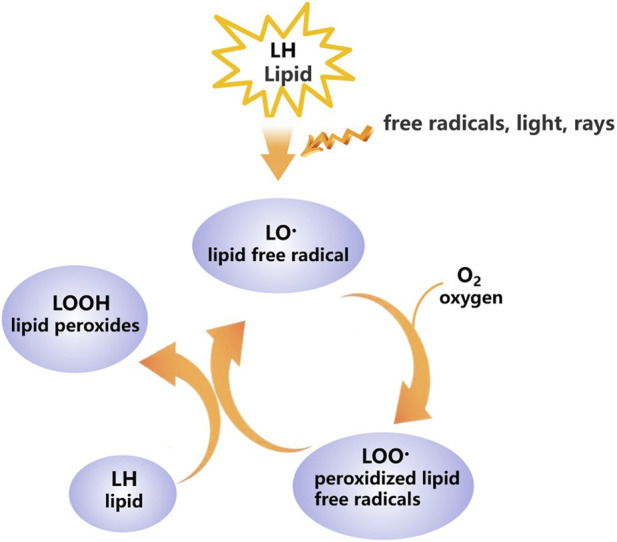
Schematic diagram of lipid peroxidation.

Lipid peroxidation is a free radical-mediated chain reaction primarily targeting polyunsaturated fatty acids (PUFAs) within biological membranes. Initiated by reactive oxygen species (ROS) such as the hydroxyl radical, it leads to the formation of lipid radicals (L·), which react with oxygen to form peroxyl radicals (LOO·). These propagate the chain by abstracting hydrogen from adjacent PUFAs, generating lipid hydroperoxides (LOOHs). The decomposition of LOOHs, often catalyzed by transition metal ions like Fe^2+^or Cu^+^, yields a plethora of reactive electrophilic species (Tschuck et al., 2024). Key terminal products include malondialdehyde (MDA) and 4-hydroxynonenal (4-HNE), which are not merely inert biomarkers but active mediators of damage through their ability to form covalent adducts with proteins and DNA, altering function and triggering secondary responses (Ya et al., 2024; Monroe et al., 2025). This self-amplifying cycle (Figure 1) can lead to exponential oxidative damage, but its site of initiation and subsequent consequences are highly context-dependent, a concept central to our framework.

### The hierarchy of molecular targets in lipid peroxidation: dictating disease specificity

A central thesis of this review is that the varied clinical manifestations of lipid peroxidation are not simply due to different end-products attacking random cellular components. Instead, pathology is initiated by a predictable hierarchy of molecular targets, determined by the unique biochemical and structural environment of each tissue. This hierarchy is governed by the intersection of local lipid substrate availability, the presence of catalytic metal ions, and compartment-specific antioxidant defenses (Zheng et al., 2024; Alves et al., 2025).

In NAFLD/Metabolic Liver Disease: The inner mitochondrial membrane phospholipid cardiolipin, enriched in PUFAs and in proximity to the ROS-producing electron transport chain (ETC.), acts as an early sentinel target. Its peroxidation precedes widespread plasma membrane damage and directly impairs ETC., complex integrity and function, leading to a vicious cycle of ROS burst and bioenergetic crisis (Aoun et al., 2012).

In Atherosclerosis: The primary initial target is extracellular: the phospholipids within low-density lipoprotein (LDL). Their oxidation generates oxidized LDL (ox-LDL), which is the key ligand for scavenger receptor-mediated uptake by macrophages, driving foam cell formation and endothelial inflammation (Koc et al., 2025).

In Neurodegenerative Diseases (e.g., Alzheimer’s): Vulnerability shifts to specific protein thiol groups, particularly in the active site of the selenoenzyme glutathione peroxidase 4 (GPX4). Its inactivation acts as a molecular switch permitting unchecked lipid peroxide accumulation, committing neurons and other cells to ferroptosis, an iron-dependent form of regulated cell death (Saraev and Pratt, 2024; Ying et al., 2025).

This hierarchical understanding moves beyond description to offer a predictive and diagnostic framework, and it fundamentally restructures our analysis. The following sections will not revisit diseases individually but will trace how these distinct initial insults—whether to mitochondrial cardiolipin, extracellular LDL, or key antioxidant enzymes—converge onto common downstream pathological themes, explaining shared pathophysiology across different organs. Detecting early, organ-specific peroxidation products (e.g., oxidized cardiolipin derivatives, specific ox-LDL epitopes) could enable earlier, more targeted interventions. It also challenges the therapeutic “one-size-fits-all” approach, suggesting that interventions must be tailored to protect or restore the relevant hierarchical target in a given disease context.

### Cross-cutting mechanistic themes in disease pathogenesis

The hierarchical initiation of lipid peroxidation sets in motion downstream cascades that converge on several cross-cutting pathological themes. Adhering to our proposed framework, we now abandon a conventional organ-system-based narrative. Instead, we reorganize the pathophysiology around three core propagation themes, demonstrating how the specific initial targets identified earlier funnel into these universal pathways, ultimately yielding organ-specific disease phenotypes.

Theme 1: metabolic dysregulation and bioenergetic crisis

Lipid peroxidation directly assaults cellular energy metabolism. This theme is a primary consequence when the initial target resides in energy-generating compartments, such as mitochondria in metabolic liver disease. In hepatocytes, as seen in NAFLD, peroxidation of mitochondrial membranes disrupts the ETC., reducing ATP synthesis and increasing ROS leakage. This bioenergetic crisis impairs insulin signaling and promotes gluconeogenesis, contributing to systemic insulin resistance (Zeng and Chen, 2022). Our work with liver-specific NPC1L1-overexpressing Bama miniature pigs provides a focused and critical illustration of this theme within the proposed framework. In this model, NPC1L1-driven SREBP activation increases hepatic free fatty acids (FFA), creating a pro-oxidant milieu with mitochondrial stress. This model crystallizes and validates the hierarchical target concept: the metabolic insult (high FFA) creates a local environment where the mitochondrial inner membrane (specifically cardiolipin) becomes the primary target. Its peroxidation, evidenced by elevated MDA and depressed SOD activity, directly drives the bioenergetic dysfunction and liver injury (Fanti et al., 2025; Xu et al., 2015). Critically, this model allows comparison with other NAFLD models (e.g., high-fat diet rodents). While the initiating metabolic driver may differ, the convergence on mitochondrial cardiolipin as a key early target and the subsequent propagation through bioenergetic crisis appear to be a shared, theme-based pathway, underscoring its fundamental role. It also highlights that targeting the initial metabolic driver or protecting mitochondrial integrity may be more effective than scavenging downstream radicals.

Theme 2: inflammatory and immune dysregulation

Lipid peroxidation products are potent instigators and amplifiers of inflammation. They function as damage-associated molecular patterns (DAMPs). This theme is prominently activated when initial targets generate pro-inflammatory ligands or neo-epitopes, as seen with ox-LDL in atherosclerosis or MDA/4-HNE adducts in various tissues. For instance, ox-LDL is not just a damaged particle but a key ligand that activates pattern recognition receptors on macrophages and endothelial cells, triggering NF-κB-driven production of pro-inflammatory cytokines (Prasad and Mishra, 2022). Similarly, MDA and 4-HNE form adducts with proteins, creating oxidation-specific epitopes that can be recognized by the immune system, potentially breaking tolerance and contributing to autoimmune responses (Jomova et al., 2025). In NAFLD, MDA recruits inflammatory cells, exacerbating hepatocyte death and fibrosis progression. Thus, regardless of the initial target (LDL in vessels or cellular lipids in the liver), the propagation through inflammatory amplification represents a universal thematic pathway that links oxidative damage to chronic, sterile inflammation, a central feature of many metabolic diseases.

Theme 3: ferroptosis and cell death decisions

The fate of cells experiencing severe lipid peroxidation is often determined by the ferroptosis pathway. Ferroptosis is an iron-dependent form of regulated cell death characterized by the overwhelming, GPX4-incompetent peroxidation of phospholipids containing PUFAs, particularly arachidonoyl (AA) and adrenoyl (AdA) phosphatidylethanolamines (Capel et al., 2020). This theme represents a final common pathway for cell loss and is directly linked to the failure of specific hierarchical targets within the antioxidant defense system, most notably GPX4. The hierarchy of targets concept is crucial here: In neurons, inactivation of GPX4 (the target) unleashes ferroptosis. In cancer cells, high PUFA membrane content and metabolic alterations can predispose them to ferroptosis (Lu et al., 2025). This theme transcends individual diseases, representing a final common pathway for cell loss in neurodegenerative disorders, certain forms of hepatocyte death in steatohepatitis, and even a potential Achilles’ heel for some tumors. Therapeutic modulation of ferroptosis—by inhibiting it in degenerative diseases or inducing it in cancer—represents a prime example of mechanism-targeted intervention.

These themes are not mutually exclusive but are highly interconnected and often co-exist, driven by the initial target. For example, in atherosclerosis, ox-LDL (initiating Inflammation Theme) can also promote endothelial cell ferroptosis (Luo et al., 2024). In NAFLD, mitochondrial dysfunction and cardiolipin peroxidation (Metabolic Theme) increase ROS, promoting both inflammation and ferroptosis-sensitivity, as evidenced in our NPC1L1 model and other studies (Aoun et al., 2012; Xu et al., 2015).

### Influencing factors and the body’s defense system

The progression of lipid peroxidation is modulated by a balance between promoting factors and the antioxidant defense system. Factors such as pro-oxidant transition metals (Fe, Cu), high PUFA substrate availability, and exogenous insults (UV, toxins) promote peroxidation. The body’s defense is multi-layered, including enzymatic antioxidants like superoxide dismutase (SOD), catalase, and GPX4, and non-enzymatic scavengers like vitamin E and glutathione (Noguchi et al., 2025). Within our framework, the integrity of these defenses directly influences the “hierarchy of targets.” A deficiency in a specific defense component (e.g., selenium for GPX4) can elevate its associated molecule (e.g., GPX4 thiols) to the top of the target hierarchy in susceptible tissues, thereby determining disease susceptibility and propagation theme. A critical, often overlooked aspect is the interdependence of these defenses. For example, vitamin E (α-tocopherol) terminates radical chains in membranes, but its oxidized form is recycled by vitamin C or systems involving GPX4 and selenium. Selenium deficiency can thus render vitamin E supplementation ineffective, highlighting the need for a systems view of antioxidant therapy (Cholewski et al., 2018).

### Nutritional and dietary influences: modulating the peroxidation landscape

Diet profoundly influences systemic susceptibility to lipid peroxidation through multiple, interconnected mechanisms. Dietary components act across our three-tiered framework: they can alter substrate availability for initial targets (Tier 1), modulate the intensity of propagation themes (Tier 2), and serve as interventions (Tier 3). Simply recommending “antioxidants” is insufficient. Key considerations include:

PUFA Balance: The ω-6 to ω-3 PUFA ratio influences membrane composition and the profile of eicosanoid signaling molecules. Higher ω-3 intake (EPA/DHA) is generally associated with anti-inflammatory and anti-peroxidative effects, partly through the production of specialized pro-resolving mediators (SPMs) (Chen et al., 2024). However, all PUFAs are susceptible to peroxidation; thus, high PUFA intake requires adequate antioxidant support.

Antioxidant Network: Dietary antioxidants (vitamins E and C, polyphenols, selenium) function as a network. Polyphenols like curcumin or EGCG may exert effects not only through direct radical scavenging but also by activating endogenous defense pathways like the NRF2-Keap1 system (Wu et al., 2011).

Pro-oxidant Load: Processed foods can deliver pre-formed lipid oxidation products and advanced glycation end products (AGEs), which directly contribute to the oxidative burden and activate inflammatory receptors like RAGE (Manoharan et al., 2024; Harlina et al., 2024; D'Cunha et al., 2022; Wang et al., 2025).

Therefore, holistic dietary patterns (e.g., Mediterranean diet) are protective likely because they favorably modulate the entire “peroxidation landscape”: reducing pro-oxidant intake, enhancing the antioxidant network, and mitigating inflammation.

### Therapeutic strategies: a critical, mechanism-informed appraisal

Evaluating interventions through our three-tiered framework reveals why some strategies have faltered and points to more promising directions.

1. Direct Antioxidants: Lessons from Failure. Broad-spectrum antioxidants (vitamin E, β-carotene) aimed at the Propagation Tier have shown limited success in chronic disease trials (Saimoto et al., 2025). Reasons include poor bioavailability, inability to reach critical subcellular sites (e.g., mitochondria), and the possibility of acting as pro-oxidants. Their role may be most relevant in prevention or in combination therapies, particularly in populations with defined deficiencies.
2. Ferroptosis Inhibitors: Targeting a Specific Cell Death Pathway. Compounds like ferrostatin-1 and liproxstatin-1 directly inhibit lipid peroxidation within the ferroptosis pathway (Theme 3). They show remarkable promise in preclinical models of neurodegeneration, liver injury, and ischemia-reperfusion (Chen et al., 2023; Xie et al., 2022; Zhao et al., 2024). This strategy exemplifies a theme-targeted intervention. The challenge is translational: developing compounds with suitable pharmacokinetics (e.g., brain penetration for neurodegenerative diseases where GPX4 is the key target) and ensuring therapeutic selectivity without disrupting physiological iron metabolism.
3. Nutritional and Lifestyle Modulation: A Foundational Strategy. This approach primarily targets the Influencing Factors, aiming to raise the threshold for peroxidation initiation. Personalized nutrition, considering genetic background (e.g., GPX4 polymorphisms) and metabolic status, is more rational than generic advice. Exercise enhances mitochondrial efficiency and endogenous antioxidant defenses (Meng and Su, 2024; Poljsak, 2011; Kruk et al., 2022).
4. Targeting Master Regulators and Upstream Drivers. This represents the next frontier, aiming at the Molecular Initiation Tier or key nodes in defense systems. Examples include:

NRF2 Activators: Boost a suite of cytoprotective genes, offering a systems-level response (Giroud et al., 2009; Hushpulian et al., 2025; Tkaczenko et al., 2025). Caveats include potential pro-tumorigenic effects with chronic activation.

Mitochondrial Protectants: Agents that stabilize cardiolipin or improve ETC., function could prevent the initial metabolic crisis in NAFLD.

Inhibitors of Pro-Oxidant Enzymes: Targeting sources of ROS like NOX4 in specific tissues can reduce the initial insult.

Our NPC1L1 model analysis within this framework suggests that inhibiting the upstream metabolic driver (e.g., intestinal cholesterol absorption or hepatic fatty acid synthesis) is a potent strategy to prevent the cascade at its origin, by removing the condition that establishes the specific target hierarchy (hepatic mitochondrial vulnerability).

Conclusion on Therapeutic Integration: Effective therapy will likely require combination strategies that concurrently: (a) reduce the initiating insult (e.g., lower FFA, sequester catalytic iron), (b) bolster specific, compromised antioxidant defenses (e.g., GPX4 support in neurons), and (c) intercept cytotoxic secondary products. Future research must prioritize the development of biomarkers that report on specific pathways activated (e.g., specific ox-phospholipids, ferroptosis signatures) to enable truly targeted and personalized interventions.

## Conclusion and future perspectives

In conclusion, this review argues that advancing the fight against lipid peroxidation-driven metabolic diseases requires a paradigm shift from descriptive biology to mechanism-prioritized, problem-solving science. By framing the issue around a central thesis—that tissue-specific target hierarchy dictates disease specificity—and rigorously analyzing the literature through a tiered framework of molecular initiation, pathological propagation via cross-cutting themes, and mechanism-informed intervention, we provide a more dynamic, unified, and actionable perspective.

Key takeaways include.

1. Specificity Matters: The hierarchy of initial molecular targets (cardiolipin, LDL, GPX4) explains disease specificity and should guide diagnostic and therapeutic targeting.
2. Themes Unify Pathogenesis: Re-conceptualizing disease progression around metabolic crisis, inflammatory amplification, and ferroptosis reveals common nodes for intervention across different organ systems.
3. Context is Crucial for Therapy: Interventions must be evaluated and designed based on the specific mechanisms dominant in a given disease stage and context. The failure of broad antioxidants underscores this point.
4. Model Systems as Mechanistic Probes: Our NPC1L1 transgenic pig model serves as a validated example of how a defined genetic/metabolic insult triggers a predictable cascade through lipid peroxidation, offering a platform for testing mechanism-based therapies.
5. The Path Forward lies in integrating multi-omics approaches to define personal peroxidation susceptibilities, developing pathway-specific biomarkers, and designing smart combination therapies that address multiple tiers of the peroxidation cascade simultaneously.

Ultimately, bridging the gap between mechanistic knowledge and clinical success will depend on our ability to move from seeing lipid peroxidation as a uniform “bad actor” to understanding and targeting its specific, hierarchical roles in the complex drama of metabolic disease.
